# Dense lying self-organized GaAsSb quantum dots on GaAs for efficient lasers

**DOI:** 10.3762/bjnano.2.39

**Published:** 2011-06-30

**Authors:** Thomas H Loeber, Dirk Hoffmann, Henning Fouckhardt

**Affiliations:** 1 Optoelectronics and Microoptics Research Group, Physics Department, Kaiserslautern University of Technology, P.O. Box 3049, D-67653 Kaiserslautern, Germany

**Keywords:** V/III flux ratio, GaSb quantum dots, growth temperature, semiconductor laser, Stranski–Krastanov growth

## Abstract

GaAsSb quantum dots (QDs) were grown on GaAs in the Stranski–Krastanov (SK) epitaxial mode. Their characteristics were dependent on the Sb/Ga (V/III) flux ratio and the growth temperature. The samples were grown with a V/III ratio between 0.45/1 and 1.50/1 and a temperature between 445 and 580 °C, not commonly used by other research groups. These parameters enabled the growth of dense lying dots with a density at least up to 6.5 × 10^10^ cm^−2^ and a diameter and height of 20 and 4 nm, respectively. The photoluminescence (PL) spectra revealed a QD peak at an emission wavelength between λ = 0.876 and 1.035 μm, depending on the exact conditions. Using a stack of such QD layers, an electrically pumped efficient QD laser was realized with an emission wavelength of λ ≈ 0.900 µm at a temperature of 84 K.

## Introduction

GaSb quantum dots (QDs) grown on GaAs wafers have received considerable attention in efforts to improve the efficiency of near- and mid-infrared antimonide lasers in the emission wavelength range between 1.0 and 1.5 μm [1]. The lattice mismatch of 7.8% between GaSb and GaAs (similar to that of InAs and GaAs) causes Stranski–Krastanov (SK) growth, depending on the precise control of the Sb/Ga V/III flux ratio, coverage, and growth temperature. Furthermore these parameters influence dot shape, size and density. The staggered (type-II) band alignment with a large valence band offset provides hole confinement. The electronic states lie in the GaAs conduction band continuum [1–2]. Bimberg et al. grew GaSb on GaAs with variable GaSb layer thickness [2–5]. Huffaker et al. achieved QDs by variation of the Sb/Ga flux ratio (V/III ratio) between 1.00/1 and 6.50/1 with a maximum dot density around 2.9 × 10^10^ cm^−2^ [1,6–9]. These samples were grown at a temperature of *T* ≈ 500 °C. So far, photoluminescence (PL) signals of SK-grown GaSb QDs on GaAs have emerged at a wavelength between 1.0 and 1.3 µm [7,9]. The interface between the GaAs buffer and the GaSb QDs can cause a wetting layer, which will lead to an additional PL peak with an even smaller wavelength around 0.92 μm [2–3,7].

In this paper we report on SK-grown QDs with a V/III ratio between 0.45/1 and 1.50/1 to find the optimum dot density and size at constant coverage and growth temperature of *T* = 527 °C. In several steps the ratio was increased from sample to sample, and the dot density was characterized with atomic force microscopy (AFM). Moreover, the PL peak of the QDs was shifted by varying the growth temperature *T* between 445 and 583 °C. For this very growth series two Sb/Ga ratios, i.e., 1.0/1 and 1.5/1, were chosen. In our case an additional peak attributed to a possible wetting layer could not be identified.

An electrically pumped QD laser was grown with an active region consisting of eight layers of QDs and an emission wavelength of λ ≈ 0.900 µm.

## Results and Discussion

### Growth procedure and dot characterization

All samples were grown on (001) GaAs substrates with an R450 molecular beam epitaxy (MBE) system from DCA Oy, Finland. The flux was determined by beam equivalent pressures (BEP) for all source materials. The partial Ga pressure was kept nearly constant between ≈1.60 and ≈1.89 × 10^−7^ hPa, for each individual growth process. Depending on the measured Ga flux, the Sb flux was set with a valved Sb cracker.

An ≈200 nm thick GaAs layer was grown as a buffer on each substrate with standard techniques at a growth temperature around *T* = 640 °C. Then, the samples were cooled down to the desired QD growth temperature of *T* = 527 °C under As flux. The GaSb QDs were grown on an As-rich surface. The nominal coverage for all samples was 3 monolayers (ML) with a growth rate of ≈0.3 ML/s.

The samples were cooled down to room temperature under the adjusted Sb flux immediately after the QD growth for topographic tapping-mode measurements with a Park XE-70 AFM and soft cantilevers. In contrast, for PL measurements the samples were capped with an epitaxial ≈90 nm thick GaAs layer without growth interruption, at the GaSb growth temperature, and then cooled down under As flux. During PL measurements, the samples could be cooled down to a temperature *T*_PL_ = 20 K and heated up in a controlled way. They were excited by a diode laser at λ = 780 nm with a maximum intensity of 70 W/cm^2^ entering the sample. Unless otherwise stated the PL measurements were performed at *T*_PL_ = 20 K.

**Figure 1:**
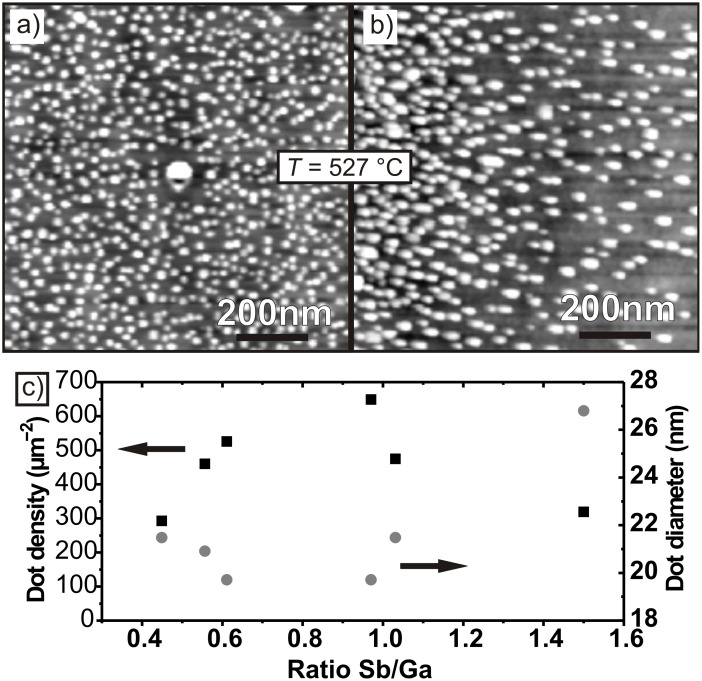
Top: AFM images of samples grown at a temperature of *T* = 527 °C and a nominal coverage of 3 ML, but with different V/III ratios: a) 0.97/1 and b) 1.5/1; c) dot density and diameter plotted against V/III ratio.

Figure 1a and 1b show exemplary AFM images of two samples both grown at the same temperature of *T* = 527 °C, but with different Sb flux. The V/III ratio was 1.00/1 for the sample in a) and 1.50/1 for the sample in b). As can be seen from the diagram in Figure 1c, with the V/III ratio increasing from 0.45/1 up to 1.00/1 the dot density increased from 2.9 to 6.5 × 10^10^ cm^−2^. (An even higher dot density of 10 × 10^10^ cm^−2^ could be achieved for a nominal coverage of 5 ML, but in this case the dots start to join at their base.)

For a nominal coverage of 3 ML, the dot shape, average height *h* = 5 ± 1 nm and average diameter *d* = 20 ± 1 nm were nearly constant for all the V/III ratios between 0.45/1 up to 1.00/1.

The dot dimensions were almost homogenous across the wafer except for a few much bigger dots (e.g., at the center of Figure 1a). The density of these bigger isolated islands was much lower (0.01 × 10^10^ cm^−2^) and thus they did not greatly affect the measurement results.

When the V/III ratio was increased further, from 1.00/1 up to 1.50/1, the dot density decreased from 6.5 × 10^10^ down to 3.2 × 10^10^ cm^−2^, while the dot height increased from *h* = 5 ± 1 nm to 7 ± 1 nm and the diameter enlarged from *d* = 20 ± 1 nm to 27 ± 1 nm. So-called smaller dots became so-called larger dots. Apparently, a V/III ratio of 1/1 is optimal for high quantum dot density. Compared to the highest QD density previously reported, our results reveal a two times higher density.

**Figure 2:**
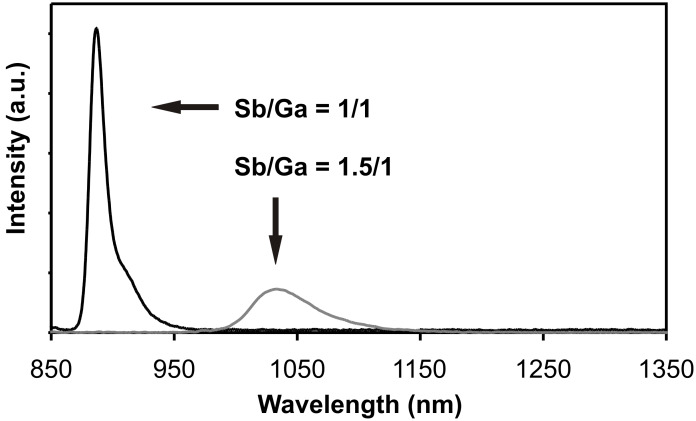
PL spectra of GaSb dots grown on GaAs with two different V/III ratios of 1.5/1 and 1/1, respectively, at a growth temperature of *T* = 527 °C and a nominal coverage of 3 ML.

Figure 2 shows the PL spectra of two QD samples grown at a V/III ratio of 1/1 (so-called smaller dots) and 1.5/1 (larger dots), respectively, at a growth temperature of *T* = 527 °C and a nominal coverage of 3 ML. The peak maximum is at λ_1/1_ = 0.887 μm with a full width at half maximum (FWHM) of 15 nm for the smaller dots. An additional peak on the right shoulder with a wavelength of 0.912 μm can be assigned to the bigger isolated islands. The larger dots emit at λ_1.5/1_ = 1.034 μm with a FWHM of 58 nm. This last peak is similar in position to those PL peaks reported in [1,6–9], [2–3,5,10] or [11–12], although the dot dimensions are larger in our case. In other publications a peak below λ = 1 μm has also been assigned to QD emission [13–16]. As seen from the difference between λ_1/1_ and λ_1.5/1_ a variation of the dot size by several nanometers, achieved via a change of the V/III ratio, already leads to a shift of the spectral position of the PL peak by ≈150 nm.

**Figure 3:**
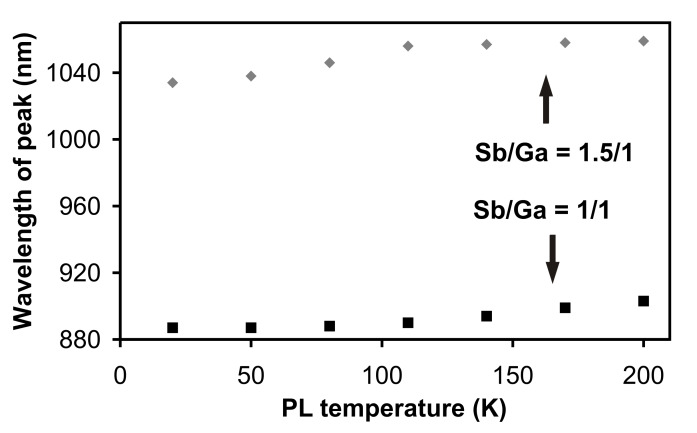
PL peak position for dots with larger diameter (at a V/III ratio 1.5/1) and smaller diameter (at a V/III ratio 1/1) plotted against sample temperature *T*_PL_.

The PL spectra were recorded at different PL sample temperatures from *T*_PL_ = 20 to 200 K in steps of 30 K. At higher temperatures no PL peak could be identified. In Figure 3 the wavelength of the PL peak maximum is plotted against the PL temperature *T*_PL_. For the smaller dots the wavelength increases slightly from 0.887 up to 0.903 µm for increasing sample temperature *T*_PL_, giving a value of 0.08 nm/K for the temperature dependent peak shift. The FWHM is almost constant at 18 ± 3 nm. The peak of the larger dots shifts from 1.034 up to 1.059 µm, giving 0.14 nm/K, with a FWHM of 75 ± 16 nm. The weaker quantization in the larger dots leads to a stronger temperature dependence. But the latter is still smaller than that of a 50 nm thin GaSb quantum well (between 100 nm thick AlAs_0.084_Sb_0.916_ barriers) with 0.35 nm/K (measured PL peak shifts from 1.533 to 1.594 µm for *T*_PL_ = 20 to 200 K). These results justify the conclusion that our dots have QD character. With increasing excitation power density up to the maximum of 70 W/cm^2^, the well-known blueshift was observed for both kinds of dots [2]. For the smaller dots the wavelength decreases from 0.893 down to 0.887 µm, while the peak for the larger dots shifts from 1.049 down to 1.034 µm.

The smaller dots have the same dimension as those mentioned by other authors, but they emit at a ≈250 nm lower wavelength [1–5,7–12]. This deviation is due to an incorporation of arsenic (As) within the GaSb QDs, due to the higher growth temperatures as compared to [10,15,17–18] and the lower Sb/Ga ratio. So the QDs actually consist of the alloy GaAs_1−_*_x_*Sb*_x_*. With decreasing Sb concentration within the QDs the emission wavelength decreased.

To estimate the actual Sb concentration within the QDs, numerical simulations were performed with the program next**nano^3^** [19]. For the calculations, the dot dimensions were taken from AFM measurements. The simulations are in good agreement with the experimental data for *x* = 0.28 ± 0.07 (GaAs_0.72_Sb_0.28_) in case of the smaller dots and for *x* = 0.52 ± 0.03 (GaAs_0.48_Sb_0.52_) in case of the larger dots. A higher Sb/Ga ratio during the growth process leads to a higher Sb concentration within the QDs and to larger dots. Both effects result in a higher emission wavelength for the larger dots.

**Figure 4:**
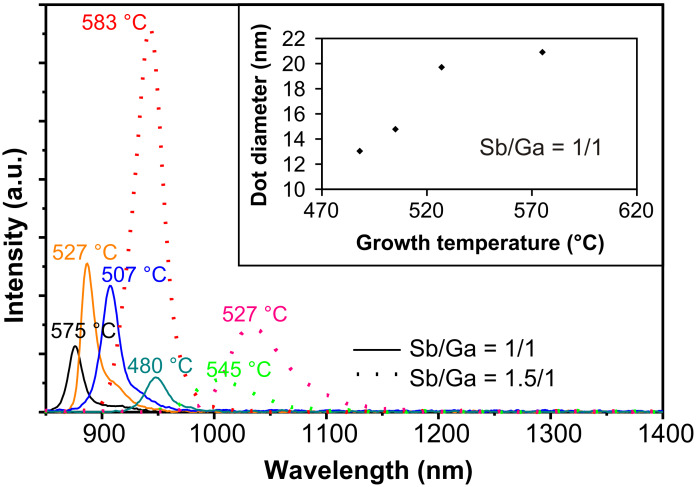
PL spectra of dots grown with a V/III ratio of 1/1 and 1.5/1, but at different growth temperatures. The PL measurements were performed at a constant sample temperature of *T*_PL_ = 20 K. Inset: Dot diameter plotted against growth temperature for samples with a V/III ratio of 1/1.

As stated above at our higher growth temperature more As can diffuse into the QDs and lead to a decrease in the Sb concentration. Our dots were grown at a temperature ≈30 °C higher than in [1–5,7–12], which could explain the blueshift of ≈250 nm. To further test this assumption, QDs were grown at different growth temperatures. The V/III ratio was kept constant at 1/1 for smaller dots or 1.5/1 for larger dots. The growth temperature was varied from ≈445 up to ≈580 °C. Figure 4 shows the corresponding PL spectra. For the samples grown at a V/III ratio of 1/1 the maximum wavelength was λ_1/1,max_ = 0.948 μm at *T* = 480 °C. No samples with higher wavelengths were observed for this ratio [20]. The minimum wavelength λ_1/1,min_ = 0.876 μm was achieved for *T* = 575 °C. Samples with a V/III ratio of 1.5/1 emitted at a maximum wavelength of λ_1.5/1,max_ = 1.035 μm for a growth temperature of *T* = 527 °C. Samples grown at a lower temperature showed no well-defined PL peak. The minimum wavelength λ_1.5/1,min_ = 0.943 μm for the larger dots was achieved at the highest growth temperature of *T* = 583 °C. This wavelength is almost equal to the maximum wavelength λ_1/1,max_ of the smaller dots. With increasing growth temperature the PL peak shifts continuously towards smaller wavelengths for both kinds of dots. Altogether the emission wavelength of the QDs can be adjusted in a wide range continuously from λ_1/1,min_ = 0.876 μm up to λ_1.5/1,max_ = 1.035 μm by changing V/III ratio and growth temperature. (With further change of the nominal coverage it should be possible to increase the emission wavelength to a value beyond 1.3 μm).

The dot diameter increased with increasing growth temperature, as shown for the smaller dots in the inset of Figure 4, in agreement with Forchel et al. [21]. The dot height also increased, while the dot density was almost constant at ≈6.5 × 10^10^ cm^−2^. The experimental PL data were confirmed by numerical simulations with next**nano^3^**, revealing a decrease of the Sb concentration with increasing growth temperature from *T* = 480 up to 583 °C. For the smaller dots the concentration dropped from *x* = 0.58 down to 0.23 (GaAs_0.42_Sb_0.58_ to GaAs_0.77_Sb_0.23_) in the mentioned growth temperature range, while for the larger dots the Sb content decreased from *x* = 0.52 down to 0.31 (GaAs_0.48_Sb_0.52_ to GaAs_0.69_Sb_0.31_).

Due to the lower Sb concentration the larger QDs emitted at a similar wavelength to those reported in [1–3,5–12], although those dots are much smaller. In other publications [13–16] QD emission as well as dimensions can be compared to our work.

According to Figure 2 an increase in the dot volume should lead to a redshift of the PL peak. However, the blueshift induced by the decreased Sb concentration in the QDs overcompensates for the volume dependent redshift, leading to the net blueshift observed in Figure 4.

### Quantum dot laser

**Figure 5:**
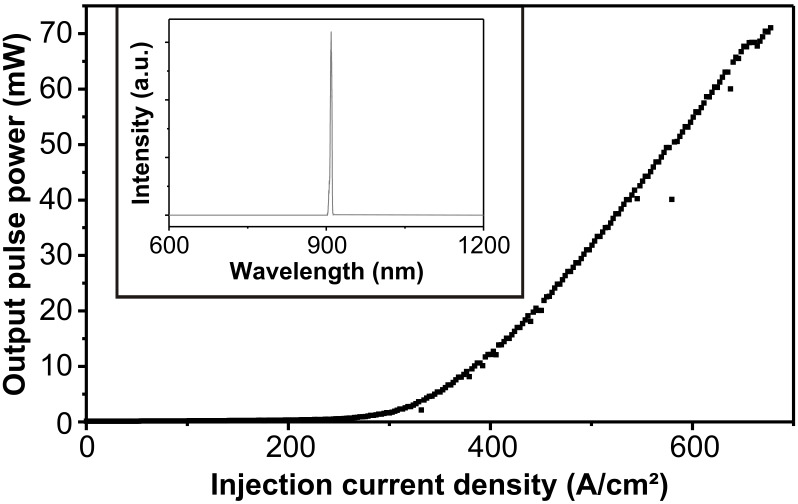
Output pulse power versus injection current density of a laser with a 1000 µm long and 30 µm wide active region, operated in pulsed mode (1 µs pulse width and a duty cycle of 0.1%) at an operating temperature *T*_Laser_ = 84 K. The threshold current density and quantum efficiency were *j*_th_ = 356 A/cm^2^ and η_d_ = 0.542, respectively. The inset shows the emission spectrum at *j* = 1.22 × *j*_th_ revealing an emission wavelength of λ_Laser_ = 0.909 µm. The cavity length and width were 1000 and 30 µm, respectively. At an operating temperature *T*_Laser_ = 84 K the laser was characterized by a threshold current density of *j*_th_ = 356 A/cm^2^ and a quantum efficiency of η_d_ = 0.542. (Lasing at room temperature was also observed, but with substantially less favourable parameters, i.e., *j*_th_ = 3924 A/cm^2^ and η_d_ = 0.03.)

The reported results on highly uniform, dense lying GaAsSb QDs allowed the realization of an efficient QD laser. The device was electrically pumped and the growth parameters of the QDs in the active region were: V/III ratio of 1/1 and a growth temperature of *T* ≈ 520 °C, leading to an emission wavelength around 0.900 µm, see Figure 5. The growth process started with a 200 nm thick n-doped GaAs buffer layer followed by a 1500 nm n-doped Al_0.50_Ga_0.50_As as lower cladding. The active region consisted of eight layers of GaAsSb QDs, separated by 50 nm thick GaAs barriers. As upper cladding a 1500 nm thick p-doped Al_0.50_Ga_0.50_As layer was then grown. The sample was capped with a highly p-doped 50 nm thick GaAs layer for optimized electrical contact.

The output pulse power versus pump current density characteristics, and the corresponding emission spectrum, are both shown in Figure 5. The emission wavelength of λ_Laser_ = 0.909 µm (at *j* = 1.22 × *j*_th_) is very close to the expected wavelength. From temperature dependent threshold current measurements, characteristic temperatures of *T*_0_ = 476.8 K for the temperature range below 140 K, and *T*_0_ = 63.2 K above 140 K, were extracted.

In principle, for different desired emission wavelengths the laser design can be kept constant, and only the QD character within the active region needs to be varied. With this concept GaAsSb QD lasers between 0.876 and 1.035 μm emission wavelength can be achieved.

## Conclusion

A very high GaAsSb QD density on GaAs of at least 6.5 × 10^10^ cm^−2^ was achieved in the SK epitaxial growth mode, with a V/III flux ratio of 1/1 at a growth temperature of *T* = 527 °C and nominal coverage of 3 ML. With increasing V/III ratio the dot size also increased. Only one PL peak was detected, attributable to the quantum dot nature; no further peak, relating to a wetting layer or otherwise, was observed. For a V/III ratio of 1/1 (smaller dots) the PL peak was at λ ≈ 0.887 μm for a growth temperature of *T* = 527 °C and a nominal coverage of 3 ML. The dots grown with a V/III ratio of 1.5/1 (larger dots) exhibited a PL peak at λ ≈ 1.035 μm and a smaller number density of 3.2 × 10^10^ cm^−2^. Numerical simulations with the program next**nano^3^** revealed a Sb concentration within the QDs of *x* = 0.28 (GaAs_0.72_Sb_0.28_) for the smaller dots and *x* = 0.52 (GaAs_0.48_Sb_0.52_) for the larger dots.

Moreover, the growth temperature had a strong influence on PL emission wavelength. With precise control of the V/III ratio and growth temperature the PL peak positions of the QDs could be varied in a wide wavelength range from 0.876 to 1.035 μm. And the blueshift induced by the increased As concentration in the QDs, due to elevated growth temperatures, overcompensates for the redshift expected for increasing dot dimensions.

Finally, an electrically pumped QD laser with an emission wavelength of 0.909 µm, a threshold current density of *j*_th_ = 356 A/cm^2^, and a quantum efficiency of η_d_ = 0.542 at a temperature of 84 K was successfully realized.
